# Integrating a Video Game Recording Into a Qualitative Research Methods Course to Overcome COVID-19 Barriers to Teaching: Qualitative Analysis

**DOI:** 10.2196/38417

**Published:** 2022-12-16

**Authors:** Nichole Stetten, Kelsea LeBeau, Lindsey King, Jamie Pomeranz

**Affiliations:** 1 Department of Occupational Therapy University of Florida Gainesville, FL United States; 2 Veterans Rural Health Resource Center North Florida/South Georgia Veterans Health System Gainesville, FL United States; 3 Department of Health Services Research, Management & Policy University of Florida Gainesville, FL United States

**Keywords:** qualitative research, pedagogy, COVID-19, video games, educational technology, web-based learning

## Abstract

**Background:**

Because of the COVID-19 pandemic, a doctoral-level public health qualitative research methods course was moved to a web-based format. One module originally required students to conduct in-person observations within the community, but the curriculum was adapted using a web-based video game exercise.

**Objective:**

This study sought to evaluate students’ perceptions of this adaptation and determine whether the new pilot format successfully met the module’s original learning objectives.

**Methods:**

Recorded footage of a video game session was used for students to observe, take field notes, and compare the results. Qualitative methods were used to evaluate student feedback on the curriculum and determine whether the original learning objectives were met. Data were analyzed using a directed content analysis.

**Results:**

The findings demonstrate that all the learning objectives of this adapted qualitative observational research assignment using a web-based video game exercise were successfully met; namely, the students learned how to compare and contrast the observational notes of peers and to evaluate how personal bias and environmental factors can affect qualitative data collection. The assignment was also positively received by the students.

**Conclusions:**

The results align with the constructivist learning theory and other successful COVID-19 implementations. Our study demonstrates that the learning objectives of a qualitative observational assignment can be addressed given that there are proper forethought and delivery when the assignment is adapted to a web-based context using a video game exercise.

## Introduction

### Background

**Figure 1:**
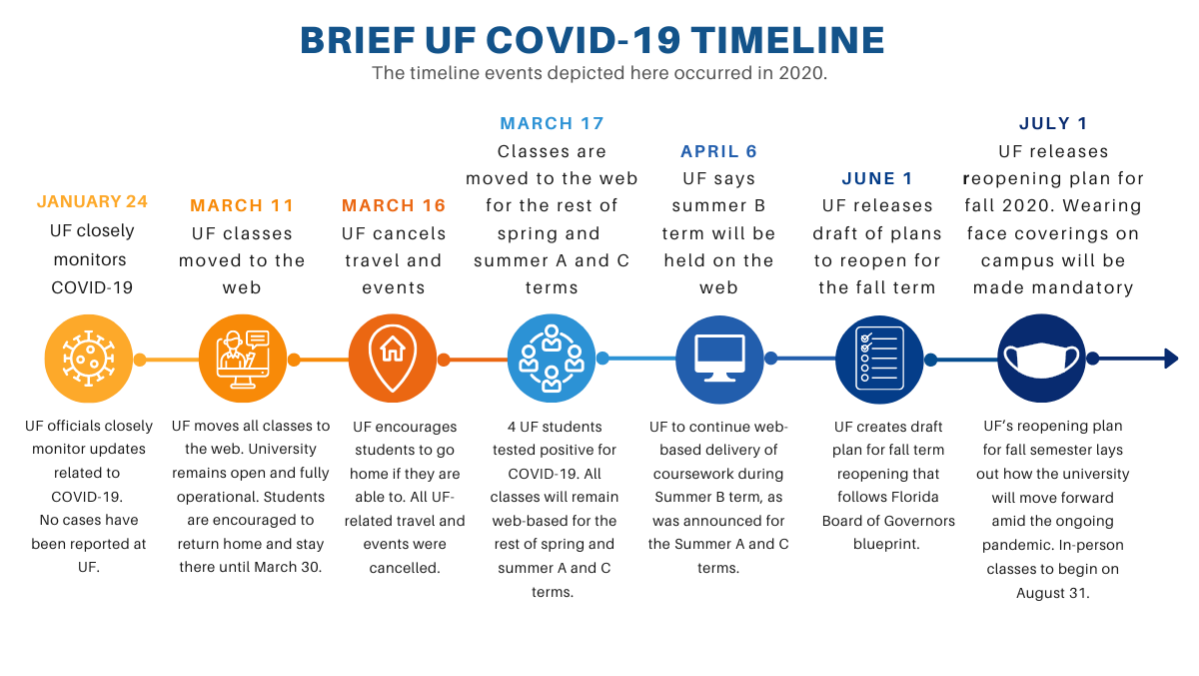
Brief University of Florida COVID-19 timeline. FL: Florida; UF: University of Florida.

On March 11, 2020, because of the COVID-19 pandemic, the University of Florida (UF) temporarily adapted all in-person courses to a web-based format. By mid-March, all classes were permanently moved to the web for the remainder of the spring semester and continued to be held this way throughout the summer semester. Courses traditionally held in person were mandated to quickly adapt to a web-based format. A brief COVID-19 timeline for UF is presented in Figure 1.

### Teaching Qualitative Research Methods on the Web

Good observation, interviewing, and communication skills are crucial for producing high-quality data for qualitative research that are reliable, trustworthy, and rigorous. These skills require a great amount of interpersonal ingenuity and are best modeled to students through in-person synchronous courses [1,2]. As colleges and universities strive to become more accessible, many courses have evolved to be taught on the web using an asynchronous format, in addition to the traditional in-person courses [1-4]. Approximately 28% of students undergoing higher education take at least one web-based learning course [5].

Teaching practices in web-based qualitative research methods have become an important topic of inquiry as opportunities for web-based and distance education increase [4]. Despite the shift to providing master’s- and doctoral-level courses on the web, there is little guidance on how to adapt qualitative research methods courses to web-based, distant formats of learning [4,6]. Specifically, there is an underdeveloped knowledge base surrounding web-based qualitative education, and existing instructions mostly emphasize face-to-face approaches [4].

### Qualitative Pedagogy and Curriculum Development

There is currently minimal direction regarding the adaptation of qualitative courses to web-based formats despite qualitative research methods comprising a core part of the curriculum for doctoral programs [6]. However, over the last few years, qualitative educators and researchers have begun building these foundations through innovative pedagogical approaches and curriculum development. Qualitative educators have applied different types of instructional strategies in web-based qualitative courses with varying degrees of success (ie, instructional media, web-based discussions, applied research activities, and writing projects) [4].

Innovative curriculums have been created using pedagogical models designed to better incorporate technology and media into the classroom, such as the ASSURE (Assure Learners, State Standards & Objectives, Select Strategies, Technology, Media & Materials, Utilize Technology, Media & Materials, Require Learner Participation, and Evaluate & Revise) model and Four Component Instructional Design model [7,8]. Additionally, 3D web-based environments have been successfully used to teach students ethnographic qualitative research methods in the web-based world of *Second Life*, in turn providing a student-centered approach to teaching and learning [3,7-11]. Furthermore, *World of Warcraft,* a massively multiplayer online role-playing game with a 3D web-based world, was used to teach a web-based doctoral level research methods course using a duoethnographic approach [6].

Developing web-based qualitative courses focuses on maintaining the same rigorous approaches and strategies as those followed in traditional in-person courses [4,10]. Commonalities among web-based courses have shown that the successful conversion of an in-person qualitative research methods course into a web-based one requires significant time and a diverse team of experts. Experts should not only be from the qualitative research field but also from the fields of instructional design and distance learning. Furthermore, adoption and diffusion of web-based qualitative research methods courses require technology innovators and early adopters [3,8,10,12]. Unfortunately, the COVID-19 pandemic has caused courses to be rapidly adapted to web-based formats, severely limiting the resources, time, and diverse professional expertise needed for teaching qualitative research methods on the web.

### Video Games and Instructional Videos as Pedagogical Tools

Researchers have shown that video games can facilitate significant learning and can be a useful pedagogical tool for instructors and educators in various academic settings [13,14], including video games designed for educational purposes (serious games) and commercial video games designed for fun and entertainment. In higher education settings, the combination of educational content and video games has been implemented to “increase student engagement” and “help achieve learning outcomes” [14]. This has been accomplished through serious games, traditional computer games, mobile games, and the modification or adaptation of commercial video games [14]. Video games in higher education have been applied to the fields of science [14,15], nursing [7,11,16], and health care leadership [9], with less application in other fields such as qualitative research and research methods.

In other research settings, instructional videos for teaching in higher education have also been shown to be an effective content delivery and pedagogical tool and cost-effective [17-20]. Instructional videos can help reimagine learning by presenting content in new ways yet offering engaging and high-value learning experiences in a web-based environment. They can also improve student learning and engagement, such as by allowing students to review or rewatch content from video recordings [17,20]. There are mixed findings regarding instructional videos because they do not always allow students to engage with the materials in the videos [21]. Creative strategies could be used to circumvent this by facilitating more active learning, such as using a video game recording where the student is following along with the video game player, figuring things out as they go, and integrating and applying the course content [17,19,21]. It is reasonable to assume that this success could be extended to video game recordings. However, knowledge regarding the use of video game recordings in higher education is lacking.

Modifying or adapting video games for educational purposes is a more recent phenomenon in the educational setting, one that continues to gain traction given that it is less expensive and resource intensive and provides instructors with a game environment that can be controlled and modified to fit the course and learning needs to students. It is important to expand the knowledge base of instructional technologies as pedagogical tools in higher education, including the modification or adaptation of video games such as video game recordings. This is especially valuable for fields that have not garnered much research attention but are central to many curriculums.

### Objectives

This study does not examine a qualitative research methods course in its entirety, as it was not developed for web-based learning. Instead, our pilot study examines how a traditional qualitative assignment that could have been assigned in person or on the web but would have been impossible to complete in person due to the COVID-19 pandemic. After considering the COVID-19 pandemic–related constraints, a web-based version of the assignment was developed using a recorded video gaming session to cultivate the competencies that the students would have acquired had the assignment been in person. The objectives of this study were to explore whether the pilot adaptation successfully met the learning objectives of the module and to explore students’ perceptions of the adapted assignment. Perceptions have been operationally defined as the students’ understandings and interpretations of the COVID-19–adapted assignment. Overall, this study hopes to add to the body of literature to support other instructors in adapting their qualitative courses to a web-based format.

### Research Questions

This study aimed to answer two research questions: (1) How were the learning objectives met after the assignment was adapted during the COVID-19 pandemic? and (2) What were the students’ perceptions of the assignment?

## Methods

### Study Design

**Figure 2:**
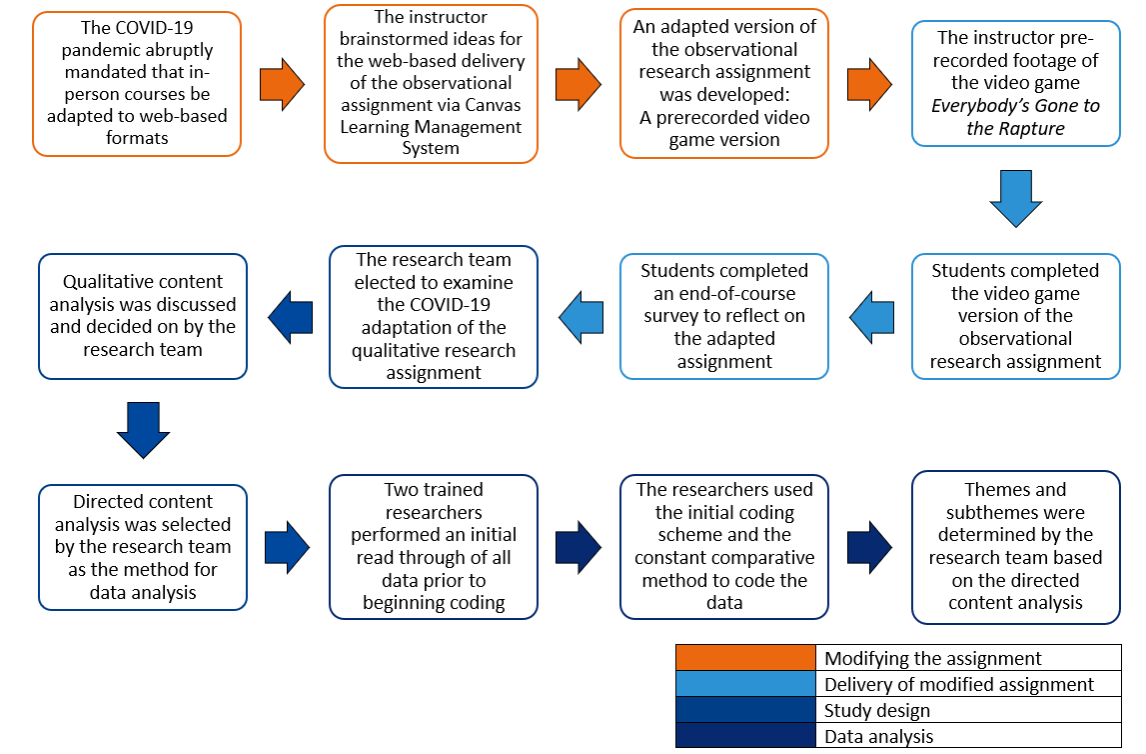
The research process and methodological decision-making.

Data were collected from a doctoral-level qualitative research methods course that included social and behavioral science public health students and rehabilitation science students. Due to the course being an upper-level doctoral course, student registration was limited to allow for deep discussions and engagements. A total of 7 students were enrolled in the study. Data were collected from an observational research assignment and students’ overall course feedback. Qualitative methods were used to evaluate students’ (N=7) course feedback and determine whether the learning objectives were met. Data were analyzed using a directed content analysis [22]. Figure 2 shows a diagram of our research process and methodological decision-making. A video game recording, an instructional technology, was selected as a pedagogical tool to deliver content and facilitate high-level learning within the web-based qualitative research methods course. The video game recording was leveraged as a learning tool to meet the educational needs of students during the pandemic while also attempting to meet the learning objectives of the qualitative research methods course. In alignment with game-based learning research [22], the goal was to create a recorded game environment that could be effectively used to develop subject-specific skills (eg, observational skills), replacing an in-person experience.

### Ethics Approval

We obtained institutional review board (IRB) approval from the UF IRB (IRB202002357). Students completed course evaluations and an evaluation of this assignment while they were enrolled in the course, and there was no research intent to use the data. Once the course was completed, the instructor applied for IRB approval to use feedback in these evaluations. Our study was approved as exempt, and informed consent was not required.

### Directed Content Analysis

A content analysis was chosen, as we sought to “provide knowledge and understanding of the phenomenon under study” [22]. A directed content analysis is used when there is prior knowledge, research, theory, or concepts that directly provide coding categories. A directed content analysis specifically follows a deductive qualitative approach, as it aims to test a specific set of learning objectives. For example, because assignments are specifically created to meet a module’s learning objectives as part of the standard curriculum development, these objectives would ultimately appear in our analysis, thus providing the initial coding categories. The initial analysis coded the text using these initial coding categories (ie, 3 learning objectives), and content that did not fit the original coding scheme was assigned a new code.

### Qualitative Trustworthiness

Quantitative research measures such as reliability and validity are not used for qualitative studies. Rather, qualitative research relies on the concept of “trustworthiness” instead of reliability and validity [24,25]. The tenets of trustworthiness are credibility, transferability, dependability, and confirmability. In this study, credibility and confirmability were used to prevent bias and improve trustworthiness. In the next section, we elaborate on how we used triangulation. To improve confirmability, 2 researchers independently analyzed the data using predetermined themes, as described in the data analysis section [24,25].

### Triangulation

Triangulation is the use of “multiple methods or data sources in qualitative research to develop a comprehensive understanding of a phenomena” [26]. Of the 5 triangulation types (ie, data, investigator, theory, methodological, and environmental), data and investigator triangulations were used. Data triangulation involves the use of different sources of data in the analysis [26]. This was accomplished by analyzing students’ answers to the assignment and course feedback (which did not directly ask students about the adapted assignment). Despite not being directly asked about the adapted assignment, most students discussed it in their course feedback. Investigator triangulation involves the use of different investigators to conduct data analysis [26]. The researchers first analyzed the data independently before coming together to compare the results. To prevent bias, the course instructor did not analyze the data. Instead, 2 researchers who were not involved in the course or assignment analyzed the data (establishing confirmability).

### Pre–COVID-19 Pandemic Assignment

During the traditional, face-to-face qualitative research methods course, students learned about different qualitative research techniques (eg, observation, interviews, and focus groups). Observation research is a qualitative technique in which researchers observe and record participants’ ongoing behavior in a natural situation. A central method used in observation research is creating observational field notes, which are descriptive, reflective, and evaluative information documented by the researcher to record observations [27]. A weakness of observational research is bias, as researchers can observe things or interpret observations differently as a result of their personal biases [27]. When teaching qualitative research, students are encouraged to practice reflexivity, wherein they reflect on and identify how their personal biases could affect data collection and analysis.

In the pre–COVID-19 pandemic assignment, to teach students about observational research and personal bias, they were asked to pair up with another student, go out into the community, and observe humans in their natural environment for a fixed period, for example, going to a coffee shop and observing the patrons in the coffee shop or focusing on a specific subset of people in that coffee shop (ie, a couple on a date, a mother and child, etc). Students were asked to observe and record observations separately and then pair up to compare the field notes.

### COVID-19 Pandemic–Adapted Assignment

Due to the COVID-19 pandemic and state restrictions, the face-to-face assignment described above was impossible to complete and needed to be adapted to the current circumstances (Figure 2). To provide an observational research experience, the instructor innovated a curriculum using a recorded video game session as a medium for the delivery and mastery of content. The instructor recorded footage of their avatar playing the video game *Everybody’s Gone to the Rapture* (Sony Computer Entertainment America LLC). In *Everybody’s Gone to the Rapture*, the player explores a small English town whose inhabitants have mysteriously disappeared. The objective of the game is to explore and discover how and why everyone has disappeared. While playing the game, the player follows around a mysterious orb of light that leads them to a scene depicting an event or interaction among the town’s occupants. These scenes, as well as other environmental cues (tape recordings, radio broadcasts, and items left by previous inhabitants), allow the player to surmise what happened. After recording 4 hours of footage, the instructor edited the video to be 30 minutes long. The 30-minute recorded video game session served as the observation environment for the students, replacing the need for in-person observations during the pandemic.

This game was chosen for the adapted assignment because it encourages exploration and careful observation. Furthermore, the game environment allowed the instructor to create highly controlled and modifiable content for the course that mirrored the face-to-face assignment in several ways. First, the observations made while watching the prerecorded footage were similar to the observations students would make while in a coffee shop. Second, both assignments required students to take observational field notes of their respective settings. To elicit similar observational experiences among the students, they were instructed to view and take observation notes on the video recording without pausing the video. This instruction was given to illicit what a live or real-time observation would feel like while taking observation notes. Finally, both assignments involved students using environmental clues and dialogue to note what they observed. The instructor had familiarity with *Everybody’s Gone to the Rapture*, which was a contributing factor to its choice for the adapted assignment. The instructor’s prior experience with the video game made it possible to knowledgeably align curriculum development and instructional goals with the game attributes, which has been recommended in educational gaming literature [6].

The students were separated into groups of 2 or 3 and asked to observe the main character in the story and how they interacted with the world while taking field notes. The students were instructed to watch the video on their own and answer several questions about what they thought was happening in it. After doing this, the students met with their partners on the web and were asked to compare their approaches to observation and taking field notes and understand how their accounts overlapped and varied. After working with their partners, they were asked to reflect on how their personal biases and current events affected their perceptions of the events in the video. The course assignment is provided in Multimedia Appendix 1.

### Student Course Feedback

At the end of the semester, the students were asked to complete an end-of-course open-ended survey and reflect on which assignment, discussion, or activity they enjoyed the most and discuss why they felt that way. The students were also asked to reflect on the aspects of the course they did not enjoy and discuss why they felt that way. This end-of-course survey was similar to the one used in another study that investigated approaches to teaching web-based qualitative research methods [10]. This open-ended survey was designed to ask students more specific questions than what was asked of them on the university evaluation system. The instructor wanted to obtain this additional feedback to learn which assignments needed to be revised or removed for future classes and which assignments should remain in the course. Data from the course feedback were included in the analysis, as 43% (n=3) of the students mentioned the COVID-19 pandemic–adapted observational assignment in their feedback.

### Data Analysis

A directed content analysis was used to investigate whether the modified assignment met the predetermined learning objectives and to understand students’ perceptions of the assignment. Directed content analysis is 1 of the 3 approaches used in qualitative content analysis and is used when the study’s frame provides initial coding categories for the study [23,28,29]. The initial coding scheme is displayed in Textbox 1 and consists of the learning objectives of the assignment. During the analysis, the data were coded by 2 trained researchers using the predetermined themes (ie, learning objectives). Data that could not be coded into the predetermined themes were analyzed and placed under new themes or subthemes. To ensure triangulation of the data, the constant comparative method [30,31] was used when organizing the data into the predetermined themes.

**The initial coding scheme used for the directed content analysis.**

**Themes**

- Learning objective 1: compare and contrast the observational field notes of peers who observed similar or the same phenomena.
- Learning objective 2: evaluate how personal bias can affect qualitative data collection.
- Learning objective 3: evaluate how environmental factors can affect qualitative data collection.

## Results

### Overview

Our directed content analysis resulted in 3 major themes directly related to the learning objectives. Subthemes were also identified during the analysis. The themes and subthemes are listed in Table 1. Students’ names and pronouns were removed from the quotes to ensure confidentiality and limit identification. Students’ names were changed to students 1, 2, 3, etc. Gendered pronouns were replaced with gender-neutral pronouns. The name Jeremy, which is used frequently by the students, is a name used in the video game recording and is not the name of a student or the instructor.

**Table 1:** Themes and subthemes from the directed content analysis.

Initial themes	Frequency, n
Learning objective 1: compare and contrast the observational field notes of peers who observed similar or the same phenomena	26
Subtheme 1.1: the web-based observational experience^a^	13
Learning objective 2: evaluate how personal bias can affect qualitative data collection	20
Subtheme 2.1: understanding one’s role in the research process^b^	6
Learning objective 3: evaluate how environmental factors can affect qualitative data collection	16

^a^Operational definition: interpretation and feedback from the students after the completion of the observational experience.

^b^Operational definition: responses that helped the students identify their role as a researcher and challenged their assumptions as they worked through the observational research assignment.

### Learning Objective 1: Compare and Contrast the Observational Field Notes of Peers Who Observed Similar or the Same Phenomena

There were 26 responses addressing learning objective 1. To accomplish this learning objective, the students observed the video game and documented field notes alone and then compared their field notes with those of a peer. After working in pairs, their observations were posted on the web on the designated e-learning discussion board, so they could compare their findings with those of other groups. By doing so, the students demonstrated an understanding of how people can observe and interpret a phenomenon in similar and different ways depending on various factors. For example, the excerpt below depicts how the perspectives of 2 students on environmental cues can overlap with one another yet also provide a new perspective, even when the assignment variables were held constant:

Accounts overlap in that we were very descriptive of environmental cues, car descriptions, etc. While another student was more narrative in [their] notes and was able to capture the audio conversations more than Student 1. Student 1 accounted more for situations surrounding the houses to account for the physical environment. Student 1 noted more subtle indications of location versus Student 6 noted final, solid indicators at the end.

Students 1 and 6

It is definitely interesting to see the different school of thoughts on exposure to the same video. Just as Student 1, I could only take short bullet point notes at a time and did not elaborate as much when typing. It is also interesting that Student 6 associated the event with rapture as I also had that as a possibility of what happened in the town.

Student 4

Students also discussed their enjoyment of the observation activity and the benefits of such an activity in a qualitative course. They emphasized the importance of recognizing that people can interpret similar events or situations in various ways:

I also enjoyed this activity as it was beneficial to understand how others interpreted the same video. The discussion solidified the differences between groups and individuals. Lastly, it reinforced the value of a team-based approach and having mechanisms in place to record your data to increase rigor.

Student 6

Student 2 and I were paired up for the observational fieldnotes assignment, which I enjoyed because it was this common stimulus and we got to experience how two researchers might approach the same dataset with different takeaways, each influenced by our experiences and biases.

Student 5

This recognition is important for qualitative researchers to ensure reliability and rigor throughout the qualitative process. Furthermore, the students discussed how the exercise of systematically comparing and contrasting their notes with those of a peer was helpful and broadened their perspectives on qualitative observational research:

We thought about participating in research on a team; even for this simple project, it was helpful to talk about what we each interpreted from the scene as that led to richer ideas and discussions. Observational notes are unlikely to be reasonably separated from the perspectives of the observer; thus, the recursive process of qualitative can help here as you can refine your ideas over time with perspectives from other researchers and from the literature.

Students 2 and 5

Overall, the comments indicated that the students perceived collaborating with peers while completing the observational research as a valuable experience, which indicated that the learning objective of comparing and contrasting the field notes of peers who observed a similar phenomenon was met.

### Subtheme 1.1: Web-Based Observational Experience

A subtheme emerged from learning objective 1 and was termed “web-based observation experience” (Table 1). This was operationalized as the interpretation and feedback from the students after the completion of the observation experience. Although this subtheme was related to the observation experience and the comparison of field notes among peers, it focused more on the comparison of the web-based observation experience with the real-world observation experience. Our analysis revealed 13 responses related to this subtheme:

If observing in real time, the observer may miss things when stopping to take notes; the perspective of making observations limits to what you can see, hear.

Students 2 and 5

The above comment could apply to a real-world observation or a web-based observation, whereas the following comments relate more to the ability to replay web-based observations multiple times:

The strategy that I used was pretty similar to yours. I tried to note the key points by jotting down notes in brief then going back later and putting them together in coherent statements. My strategy differed from yours because I treated the video as a recording that I could replay (I watched it twice and I used the pause command the second time through to allow time to jot down detailed notes like the time on the clock tower for example).

Student 2

Recording would be very helpful in research involving observations. This would give the opportunity to observe multiple times and not have to focus on taking detailed notes in the first viewing.

Students 3, 4, and 7

Both examples relate to how note-taking, whether performed while observing in person or on the web, may not allow a researcher to capture the full observation if the ability to record is not available. As student 4 stated, you “need more than one perspective present to compare and contrast what you observed.” Overall, this demonstrated that students developed a deeper understanding of the strengths and limitations of observational research, especially those related to learning objective 1.

### Learning Objective 2: Evaluate How Personal Bias Can Affect Qualitative Data Collection

The qualitative analysis of learning objective 2 yielded 20 responses related to personal bias affecting qualitative data collection (Table 1). The students discussed how their personal biases impacted their observations in ways that they were not aware of until they talked with their peers. For example, this student discussed how their public health background predominantly influenced their observations and interpretations, whereas their peer’s observations and interpretations were heavily influenced by their religious beliefs:

I think my personal bias affected my observations in ways I did not know until my discussion with Student 6. They saw an element of religion and supernatural within the story, predominantly the role and symbolism of the orb. I am not a very religious individual, therefore, I relied on my public health background, which made me omit or neglect any signs of religion. I was only made aware of religion at all when scripture was recited to Jeremy [character in the game]. I think a very obvious bias was my assumption that he was a dad versus a religious leader. I think my background of science and rigor made it hard for me to be creative and open to a fictional situation, so I was trying to make connections within my reality, not the reality of the game.

Student 1

Several students also commented on their prior experience or lack of prior experience with video games and how that may have impacted the data collection:

I don’t have much exposure to modern video games, so it’s interesting to hear how experience with this type of “world” seems very helpful in navigating/interpreting a video like this. Coupled with Student 2’s discussion of [their] electronics background and how it helped them interpret the video, I think this is a great illustration of how our own experiences and perceptions can influence our observations and interpretations.

Student 7

Many also commented on how their experiences with movies or books could have also influenced the data collection process:

I think horror movies, sci-fi movies (you mentioned you are reading a sci-fi book), and video games made me think aliens or something supernatural. One aspect that really connected me to aliens was actually the choir voices that were heard throughout [background music within the game]. In the video game “Halo,” a similar ominous choir sound is heard, and it involves aliens. This exercise was a great example to demonstrate how our own personal bias can construe how we observe the world, for me, I think that went along with horror movies (specifically, zombie movies) where characters just need to survive in post-apocalyptic world. Exploring how your backgrounds/personal biases may have impacted how you viewed the video!

Student 3

I realized my thoughts on what had happened were influenced by my love for sci-fi and horror series and movies. Similar events tend to be portrayed in such movies and I was quick to form theories or assumptions that were highly correlated with the content I had previously seen.

Student 4

The students demonstrated their ability to critically evaluate how personal biases, such as backgrounds and experiences, affected their qualitative data collection process. Based on their personal biases, the students processed how their approach to data collection varied and how this variation helped their overall comprehension of personal bias in research.

### Subtheme 2.1: Understanding One’s Role in the Research Process

The subtheme understanding one’s role in the research process emerged from our analysis under learning objective 2. This subtheme was operationalized as responses that helped the students identify their role as a researcher and challenge their assumptions as they worked through the assignment (Table 1). A total of 6 student responses emerged.

Several students discussed how they struggled to determine what their role was within this assignment:

We made assumptions at the beginning that we eventually changed, which is seen throughout our notetaking. We struggled to identify who we were in this story, so we continually checked ourselves and our role. Are we part of the story or are we an external observer? We also realized the time-extensive nature of data collection and we only feel like we have a piece of the story. The notes left us with more questions than solutions, which may parallel how research questions are developed.

Students 1 and 6

Student 7 also discussed how they struggled to determine their role in the game:

Like Student 1 mentions, I initially assumed I was Jeremy [character in the game], once it became clear I wasn’t Jeremy, I had the understanding that we are a first-person observer, as you describe. However, even as Jeremy I felt that I was trying to figure out what was happening/had happened in the village. While much of my interpretations of the light and its effect were similar to what you (Student 5) and Student 2 describe, it didn’t occur to me that the light could also be functioning as a guide for the observer.

Student 7

### Learning Objective 3: Evaluate How Environmental Factors Can Affect Qualitative Data Collection

For our third learning objective, the directed content analysis yielded 16 student responses. Based on their own experiences and other students’ discussions of their respective experiences, the students contemplated whether certain environmental factors affected their data collection process and how this occurred during the observational assignment. Several students discussed how the COVID-19 pandemic directly or indirectly affected their perceptions of this assignment:

Seeing the quarantine signs immediately triggered a parallel to today’s pandemic. My mind went to a virus outbreak and maintained that position through the entire narrative. I think this kept me from deviating to religious or supernatural explanations. Though the pandemic was subtlety influencing my perceptions, I think my personal biases and experiences were more dominant in influence. I was able to make connection such as closed businesses and required quarantines, but the differences in symptoms and apocalyptic state of the community was different enough for me to separate today’s reality from the virtual one.

Student 1

I definitely think being in the middle of the COVID-19 pandemic impacted my perception of this video; I think this may have heightened my sensitivity to the term “quarantine” and its possible role in the events in the village, particularly in conjunction with the quarantine signs. I initially made the connection between the term quarantine and illness, and I assumed something had “infected” the individuals living in the village. This doesn’t necessarily align with all of the other observations in this video…but it was consistently in the back of my mind while I watched the video and tried to piece together what might have happened.

Student 7

The ongoing pandemic made it easier for me to conclude that some form of infection spread through the town and led to people quarantining. The town had a number of quarantine signs posted on doors. It was easier to reach this conclusion after experiencing the implications of the emergence of COVID 19, unlike if I had not experienced this.

Student 4

Student 3 indicated an indirect effect of environmental factors on their data collection experience. At first, they did not think that the COVID-19 pandemic influenced their data collection. However, upon discussing and evaluating other students’ responses to environmental factors, student 3 noted that they were “sensitive to the word ‘quarantine’” throughout the assignment.

In addition to environmental factors affecting qualitative data collection experiences, the students also expressed that relevant environmental factors (ie, the COVID-19 pandemic) impacted their perceptions of the observational assignment:

I think that events surrounding COVID-19 affected the way I perceived what was going on in the video because I felt that the facilitators of this course would piggyback on current events to illustrate a point with regard to this module of instruction. It was a valuable experience in terms of recognizing subjective biases and controlling for them to enhance the likelihood of an objective exploratory exercise.

Student 2

Students exhibited their abilities to evaluate the influence their immediate environments might have on qualitative data collection. This was especially apparent when students discussed how the COVID-19 pandemic directly affected what they paid attention to in their web-based surroundings, which environmental cues most impacted their interpretations, and how they perceived what was happening through documented observational field notes.

## Discussion

### Principal Findings

Due to UF’s mandated transition to web-based learning (Figure 1), it was necessary to create qualitative research assignments that could be implemented in a web-based environment. This study used learning objectives and student perceptions to examine the effectiveness of using a video game to replace a traditional in-person observational research assignment during the COVID-19 pandemic. The pilot COVID-19–adapted assignment successfully met all learning objectives. Although the learning objectives for in-person courses do not always readily conform to web-based contexts [32], our study demonstrates that learning objectives can be addressed given that there are proper forethought and delivery method. Even within the confines of rapidly adapting the observational assignment to a web-based context, our findings show that a web-based observational assignment can meet learning objectives.

### Learning Objective 1

Our findings demonstrated that the students learned how to compare and contrast their observational notes with those of their peers who observed the same or similar phenomena through the video game assignment. Students were able to compare not only note-taking styles but also the level of detail recorded. As the students viewed the same prerecorded video game scenes, it was easy for them to identify how their observations were similar and how they differed. The students reported noticing key differences among observers and identifying the importance of comparing and contrasting field notes, showing that learning objective 1 was met through the web-based assignment.

This activity also reinforced the foundational concept of analyzing qualitative data using a team-based approach. This team-based approach begins to teach students the importance of triangulation in qualitative research, which helps ensure the validity of the results [26,33]. The students were provided first-hand experience with investigator triangulation, which occurs when ≥2 researchers observe a specific phenomenon. Multiple observations allow researchers to confirm and add breadth to findings through different perspectives.

The students’ observations and notes were not just contained within pairs but also displayed on a discussion board so that the students could compare them among their classmates. This allowed the students to experience data source triangulation, which occurs when data are collected from different individuals with varying experiences and beliefs to gain multiple perspectives [26]. As the students were from different doctoral programs (rehabilitation science and social and behavioral science), they were trained to notice and see things from varying perspectives. In future courses, this can also be used to teach students additional factors that should be considered when conducting observational research (ie, ethics, consent, natural observation, laboratory or simulated observations, and equipment needed for recording).

### Learning Objective 2

The findings also demonstrated that learning objective 2 was met through this assignment. Students learned to evaluate how personal biases can affect qualitative data collection. A core aspect of conducting qualitative research is reflexivity. Reflexivity is an awareness of the influence the researcher has on the environment and the people being studied as well as how the research affects the researcher [34,35]. A large portion of being reflexive is understanding how one’s personal biases affect how one observes the world. After the students began to compare their findings with those of other students, they became aware of their biases and how their viewpoints were influenced by a particular lens. The students who had religious backgrounds noticed more religious symbolism than the students who were not religious. Some students with public health backgrounds interpreted events through more of a public health lens. Other students perceived events of the game as something supernatural—similar to a video game they had played, movie they had seen, or science fiction book they had read. The ability to identify one’s biases and evaluate how those biases shape one’s observations is important in any type of research, and it allowed the students to further understand the role they play in the research process. The students reporting their biases and reflecting on how their biases influenced their qualitative approach to the assignment demonstrated mastery of the skill identified through learning objective 2.

### Learning Objective 3

There is plenty of research showing how environmental factors affect behaviors, but these factors can also impact data collection and analysis. *Everybody’s Gone to the Rapture* displays signs of a quarantine as well as shows environmental cues that the residents were ill (tissues, blood, and dialogue between the residents about them being sick and worried). As the students were observing this within the game, in their real lives, they were surrounded by COVID-19. The students described how their minds made parallels to the ongoing COVID-19 pandemic, and them reflecting on how the world’s current events influenced their perceptions, which showed that learning objective 3 was met.

### Student Perceptions

At the end of the semester, the students mentioned this assignment as one of their favorite assignments for the course. Not only was it a change of pace for the students, but it also allowed them to work together on the web during a time of social isolation. During the prepandemic period, university students experienced high rates of depression and anxiety [36,37]. The COVID-19 pandemic and other world events (ie, wildfires, natural disasters, political unrest, etc) have caused a significant increase in depression and anxiety among students [38,39]. The students mentioned that they enjoyed working with other students via the web during the assignment. This highlights the importance of interactive assignments that allow students to connect with their peers when learning on the web [40] not only to reduce social isolation but also to create a sense of community within the classroom.

### Theoretical Alignment, Qualitative Pedagogy, and Curriculum Building

Although the entirety of this study was not directly informed by the constructivist learning theory, our results suggest that the assignment aligns with the constructivist philosophy and teaching methodology of active learning through real-world experiences (ie, experiential learning) and student-centered approaches [9,11]. Constructivist environments and the associated approaches to learning allow students to obtain knowledge, master new skills, challenge assumptions, re-examine beliefs, and collaborate with classmates to make new connections and gain different perspectives [9,11,40]. Although the real-world setting was modified for a web-based learning environment, the thoughtful modification of our assignment allowed students to directly engage in active-learning experiences and times of reflection, and it encouraged the students to work through observational research processes as they completed the assignment.

Constructivist learning strategies also encourage multiple perspectives to represent the topic area of interest. The observation assignment had a discussion portion in addition to the video game portion. Franco and DeLuca [9] argued that group interactions and discussions are the elements required for success. The discussion required students to reflect upon their personal experiences observing the video game recording and present their reflections to their classmates to compare their experiences. Their classmates’ approaches to observation techniques, as well as their own interpretations and understandings, were presented so that the students developed a well-rounded comprehension of observational research and field notes. In line with constructivism and experiential learning, this assignment provided the students with a self-directed learning environment and the opportunity to collaborate with their peers [9,11]. The assignment improves upon standard pedagogy by increasing student motivation and interaction through active-based learning and storytelling elements.

### Comparison With Prior Work

Although the literature is limited because of the novelty of COVID-19 pandemic, our study and its findings align with the recently published literature concerning COVID-19 and teaching higher education [32,40-42]. With the implementation of our video game assignment, we intentionally adapted a traditional in-person assignment to reflect the activities students that would have experienced if the class were held in person. The assignment was adapted to provide students with a meaningful and interactive experience, which has been encouraged by researchers during the pandemic [32]. This study adds to the current COVID-19 literature by providing ideas, success stories, and lessons learned regarding adapting and reimagining an in-person qualitative research methods assignment to a web-based learning environment.

Outside the scope of COVID-19, our study adds to the literature on qualitative research. Research is sparse regarding the facilitation and implementation of web-based qualitative research methods courses [4,10]. We provide an example of a web-based qualitative research methods assignment, but the scope of this study goes beyond this assignment. Due to the pressure of the COVID-19 pandemic to rapidly adapt to web-based learning, our study helps demonstrate how this can be done while still meeting learning objectives. This study can be used as a starting point for instructors who wish to adapt qualitative courses for web-based learning or for instructors who have been forced to do so given the pandemic-related constraints.

In their rapid communication opinion piece, Neuwirth et al [32] argued that in the face of the COVID-19 pandemic, higher education instructors should be adapting and preparing for a “new normal” as opposed to a “return to normal.” That is, the uncertainties that come with the pandemic should lead instructors to “re-envision” and “reimagine” how curriculums are designed and delivered to students [32]. We can adapt by embracing the new normal instead of resisting it. Our findings demonstrate that our video game observational assignment is consistent with this call to action. Furthermore, the observational assignment incorporated aspects of student engagement, interaction, and peer-to-peer instruction (ie, individual and group aspects and discussion boards for students to compare their experiences and observations), which are both important features (student engagement and interaction and peer-to-peer instruction) in a web-based environment [11,32,40]. These features allowed students to further engage with others and the assignment, solidifying their knowledge and skills for observational research.

Neuwirth et al [32] also stressed the importance of considering the economic and pragmatic burdens or constraints students face because of the pandemic. These burdens and constraints can realistically impact students’ abilities to fulfill class requirements. Pandemic constraints were hastily considered when reimagining the format of the observational assignment. From a pragmatic standpoint, we realized that we could not require the students to venture out into a public area to conduct their assignment. We also realized that economic constraints must be considered, which was one of the reasons why the assignment was prerecorded. The recorded video game segment was of no cost and required no additional software to be purchased. Thus, efforts were made to circumvent pandemic constraints.

We demonstrated how a web-based method for delivering a qualitative assignment can be successfully implemented in a qualitative research methods course to meet learning objectives, providing additional support for such an assignment. This aligns with a study that investigated the use of *Second Life*, a 3D web-based environment, for teaching qualitative research methods. As part of this study, students conducted web-based observations and interviews via the *Second Life* platform as a simulation for conducting ethnographic research [10]. The results indicated *Second Life* is an innovative approach to teaching qualitative research methods, especially regarding a qualitative course delivered on the web. Two recently published articles have presented similar experiences with the web-based delivery of educational escape room assignments. Both articles found that the learning objectives for their classrooms were met when using digital escape rooms to teach concepts, such as problem-based learning, and that they (the two articles' instructional designs mentioned earlier in the paragraph) “promote engagement, active learning, and teamwork” [41,42]. They also found that these strategies for digital instruction can use student-centered approaches [41]. Web-based methods for assignment delivery are ideal for the current COVID-19 learning environments.

### Lessons Learned

Although our study only focused on only one of the aspects of our course, we would be remiss not to include the lessons learned from our overall experiences with converting this face-to-face qualitative research methods course into a web-based course. As many institutions spend large portions of their curriculums focusing on quantitative research methods, students are often overwhelmed by an entirely new way of critical thinking that encompasses qualitative research. Students desire more synchronous opportunities to discuss readings and assignments and practice data analysis. This interaction can easily be added to a web-based synchronous qualitative course using Zoom videoconferencing (Zoom Video Communications), Microsoft Teams (Microsoft Corp), etc. This study presents numerous opportunities within the realm of qualitative teaching pedagogy, including improvements to the assignment examined in this study. In fact, the instructor has begun to adapt the course from the lessons learned while teaching on the web during the pandemic.

Readings for qualitative research can be dense and difficult to understand, especially when learning qualitative methods first. For future courses, the instructor will post readings and other material on the Perusall learning platform, allowing students to add comments and questions to readings, and see and respond to students’ responses [43]. Perusall also allows instructors to see where students spend the most time reading and provides statistics on areas of the material that students may not grasp. This allows instructors to elaborate on and expand on these areas. Adding this feature to the course directly gives students additional opportunities to engage in and discuss material.

Discussion board assignments in this course did not receive positive feedback from the students, except for the observational research assignment described in this study and another assignment that gave the students data to practice coding (qualitative data analysis). Both activities show that the students appreciate discussion boards that allow them to acquire “hands-on” experience with qualitative research methods and then partner with other students to discuss and reflect on what they have learned. Furthermore, the students’ responses suggest that interaction with the instructor and fellow students on discussion boards is important [40]. Qualitative courses should incorporate opportunities for students to acquire hands-on practice in different aspects of qualitative research.

### Limitations

The current limitations of this study include its small sample size. This course is an advanced-level doctoral course; therefore, small class sizes are typical. Second, as data were collected from an upper-level doctoral course, the results may not be applicable to qualitative courses whose curriculums are developed for undergraduate students. Although this assignment was developed for doctoral students, we believe that it would also be accessible to undergraduate students with minor adaptations.

### Conclusions

Research on the delivery of qualitative research methods courses using web-based learning environments is still in its infancy. We explored whether a pilot web-adapted video game qualitative observational research assignment successfully met the learning objectives of the qualitative research methods course and examined the students’ perceptions of the adapted assignment. While our study provides insights into 1 adaptation of a qualitative assignment, it demonstrates that web-based environments can be used to meet qualitative research learning objectives and adds to our understanding of pedagogical practices for the web-based delivery of qualitative research methods courses. Furthermore, we demonstrate how the assignment aligns with the constructivist learning theory and other successful COVID-19 implementations. With the current pandemic and the resulting digital transformation, it is important to develop methods for an engaging, efficient, and effective delivery of qualitative content and the mastery of qualitative skills without relying on traditional in-person techniques.

## Appendix

Multimedia Appendix 1 COVID-19 pandemic–adapted assignment.

## Abbreviations

ASSURE: Assure Learners, State Standards & Objectives, Select Strategies, Technology, Media & Materials, Utilize Technology, Media & Materials, Require Learner Participation, and Evaluate & Revise
IRB: institutional review board
UF: University of Florida
