# Supplementary material for: Integrating a Video Game Recording Into a Qualitative Research Methods Course to Overcome COVID-19 Barriers to Teaching: Qualitative Analysis
Source: JMIR Serious Games. 2022 Dec 16;10(4):e38417. doi: 10.2196/38417 (PMC9762141; doi:10.2196/38417)
Supplement: Multimedia Appendix 1 [file games_v10i4e38417_app1.docx]

# Appendix A: COVID-19 Adapted Assignment

This is discussion is an observational field notes exercise. Part of this discussion you will complete on your own and the other part you will complete with a partner.

**Part A:**

Watch the video below. While you are watching the video take notes about what you are observing and hearing. The video that you will be watching comes from a video game after "an event" has occurred. I have edited out portions of the video, so sometimes you will jump from one location to another.

[Click here for video link (Links to an external site.)](https://mediasite.video.ufl.edu/Mediasite/Play/e786da35286c4b7897062e546f63238b1d)

**Part B: (10 points)**

After watching the video answer the following questions:

1. What were your key observations from this video? Share your field notes. After looking over your field notes, respond to the questions below:
2. What do you think was happening in the video you just watched?
3. Who is Jeremy in this story?
4. What do you think happened to the people in this town?
5. How is the light a part of the story?

**Part C: (10 points)**

After you have taken your notes, connect with your partner via Zoom (pairings listed below) and share your notes with each other. Using each other’s field notes discuss the following questions”

1. How did your accounts overlap and/or differ?
2. How did your approaches to observation and notetaking differ (e.g., style, focus)?
3. What does this exercise teach you about observation?
4. What does this exercise teach you about fieldnotes?
5. What other specific questions or ideas did this experience raise about the research process?

**Part D: (5 points)**

After working with your partner. Answering the following questions on your own:

1. How do you think personal bias affected the way you saw things during the observation?
2. How do you think the current events surrounding COVID-19, affected the way you perceived what was going on in the video?

**Part E: (5 points)**

Post parts A through D in the discussion post (one post per group). After you have completed the discussion, look at two other groups, and comment on their observation notes, reflecting on how they saw things similar or different than you did.
